# OSMR induces M2 polarization of glioblastoma associated macrophages through JAK/STAT3 signaling pathway

**DOI:** 10.3389/fonc.2025.1538649

**Published:** 2025-03-14

**Authors:** Changcheng Xiao, Liming Tan, Xiaofei Liu, Min Zhou, Ping Chen, Zhao Wang, Bing Wang

**Affiliations:** ^1^ Department of Neurosurgery, The Second Affiliated Hospital, Hengyang Medical School, University of South China, Hengyang, Hunan, China; ^2^ Department of Neurosurgery, The Second Xiangya Hospital of Central South University, Changsha, Hunan, China

**Keywords:** OSMR, glioblastoma, JAK/STAT3 signaling pathway, macrophage, M2 polarization

## Abstract

**Introduction:**

Verify whether Onconstatin M receptor (OSMR) plays a regulatory role in the growth of glioblastoma (GBM) and explore its specific regulatory mechanism.

**Methods:**

*In vitro* investigations were carried out using OSMR knockdown and treatment with JAK agonist Butyzamide (JAKa). Evaluate cell proliferation rate through CCK-8; Colony formation experiment to detect cell proliferation; Transwell experiment evaluates cell invasion; Cell scratch assay to detect cell migration; WB detects the expression levels of pathway related proteins JAK, p-JAK, STAT3, p-STAT3, and CCL-2; Flow cytometry analysis of apoptosis rate, cell cycle arrest rate, and proportion of M2 macrophages; RT-qPCR was implemented to identify the expression of M2 polarization factors CD206, CD163 and IL-10 in macrophages. In the *in vivo* experiment, SF188 cells were subcutaneously injected into mice’s right sides and divided into two groups: those with knocked down OSMR or those without. The knocked down OSMR group was divided into subgroups treated with DMSO containing or not containing JAKa. Subsequently, the tumor volume and weight of the mice were measured. RT-qPCR was utilized to assess the level of M2 polarization-related components in tumor tissues, while flow cytometry was employed to determine the M2 polarization ratio of macrophages in tumor tissues.

**Results:**

Knocking down OSMR dramatically reduces tumor cell proliferation, invasion, and migration, accelerates cell death and cell cycle arrest, and lowers JAK and STAT3 phosphorylation as well as CCL-2 expression levels, all while decreasing the fraction of M2 macrophages. Furthermore, knocking down OSMR drastically lowered tumor development and M2 polarization levels of monocytes in tumor tissue. JAKa reversed the inhibitory effect of OSMR knockdown on GBM malignant development and macrophage M2 polarization in both *in vitro* and *in vivo* studies.

**Conclusion:**

OSMR promotes the JAK/STAT3 signaling pathway, which promotes malignant glioblastoma growth and macrophages M2 polarization.

## 1 Introduction

Glioblastoma (GBM) is the most frequent primary malignant tumor in the central nervous system, with poor treatment outcomes, high recurrence rates, short survival, and a mean duration of survival of about 8-15 months (1, 2). The high heterogeneity, strong radiation resistance, rapid proliferation ability, and high invasiveness of GBM (3, 4) make it one of the deadliest cancers. After treatment with radiotherapy, surgery and chemotherapy, GBM patients’ survival rates remain dismal (5–7). Therefore, exploring how to inhibit the malignant behavior of GBM cells and promote their death has become a key focus in the treatment of GBM.

There are abundant macrophages in the microenvironment of GBM (8), accounting for 30-50% of glioma tissue (9). Macrophages are immune cells derived from bone marrow, divided into M1 and M2 types, and can infiltrate tumor tissue under the influence of chemotactic factors secreted by glioma cells, exerting effects on tumor tissue (10–12). M1 has an inhibitory effect on tumor tissue, while M2 has a promoting effect on tumor tissue (13, 14). However, the specific mechanism of macrophage polarization in GBM has not been clearly studied.

Onconstatin M receptor (OSMR) is a member of the interleukin-6 receptor family that is predominantly associated to the JAK/STAT3, ERK, and PI3/Akt signaling cascades (15). It has the ability to modulate cancer cell invasiveness, metastasis, angiogenesis, and viability (16–18). Some studies have shown that the expression of OSMR increases in cancer (19), and it can promote tumorigenesis in solid tumors such as breast cancer (20), pancreatic cancer (21), endometrial cancer (17), cervical cancer (18), and ovarian cancer (22). Geethadevi et al. discovered that the activation level of OSMR is mostly controlled by autocrine and paracrine OSM, which are mainly produced by tumor associated macrophages (TAMs) (22). Therefore, the regulation of OSMR and macrophage polarization may play a significant role in cancer.

The JAK/STAT3 signaling pathway is a crucial signaling pathway for cytokine activation, with crucial roles in a variety of biological processes (23), mainly activated by IL-6 receptors (24). The JAK/STAT3 signaling pathway has been found discovered to be activated in a number of malignancies (25, 26), particularly colorectal cancer playing an important part in maintaining the tumor microenvironment (27). Yang et al. observed that the JAK2/STAT3 signaling pathway can induce M2 polarization in macrophages (28).

Overall, this study established the particular role and possible mechanism of OSMR in GBM through *in vivo* and *in vitro* dual level tests that knocked down OSMR expression in GBM. This will provide new therapy options for GBM.

## 2 Materials and methods

### 2.1 Cell culture and processing

Human monocytic leukemia cells THP-1 (IM-H260), human astrocytes (IMP-H223), and human glioblastoma cell lines SF188 (IM-H464) and U251 (IM-H421) were cultured in their respective specialized media (IM-H260-1, IMP-H223-1, IM-H464-1, M-H421-1) under 37°C and 5% CO_2_ conditions. All cells and professional culture media were purchased from Xiamen Immocell Biotechnology Co., Ltd. Cultivate THP-1 cells to a confluence of 70-80% and add 100 ng/mL Phorbol 12-myristate 13-acetate (PMA, HY-18739, MedChemExpress, New Jersey, USA) for 48 h to differentiate THP-1 cells into macrophages.

Astrocyte, SF188 and U251 were used for OSMR expression validation, and glioblastoma cell lines with high OSMR expression levels were selected for subsequent experiments. Subsequently, perform the following processing on it:

1. sh-OSMR screening and processing: Cells were randomly distributed into two groups: sh-NC group and sh-OSMR group. When the cells were cultured to a confluence of 70-80%, sh-OSMR lentiviral particles (sh-OSMR 1#, sh-OSMR 2#, sh-OSMR 3#) and negative control lentiviral particles sh-NC were added for infection. After 48 h of infection, RT-qPCR and WB detection were performed to screen for the best knockdown effect of sh-OSMR for subsequent experiments. All slow viruses were purchased from VectorBuilder.
2. JAK agonist treatment: Cells treated with sh-OSMR were randomly distributed into two groups: sh-OSMR+DMSO group and sh-OSMR+JAKa group. The cells were cultured to a confluence of 70-80% and treated with an equal amount of Dimethyl Sulfate (DMSO, D2650, Sigma Aldrich, St. Louis, MO, USA) containing or not containing 3 μM JAK agonist Butyzamide (JAKa; HY-148748, MedChemExpress, New Jersey, USA) for 15 min. Subsequently, a new culture medium was replaced and transferred to the upper chamber of the Transwell co culture system (29). An equal amount of THP-1 derived macrophages were added to the lower chamber and co cultured for 48 h before conducting subsequent experiments.

### 2.2 CCK-8

The proliferation of cells was detected using the CCK-8 test kit (HY-K0301, MedChemExpress, New Jersey, USA). Inoculate the processed cells (1 × 10^3^/well) onto a 96 well plate and incubate for 0, 24, 48, and 72 h. Discard the culture media and wash the cells with PBS. Then, culture at 37°C and 5% CO_2_ with 10 μL CCK-8 solution and 90 μL DMEM medium for 2 h. Measure the OD value at a wavelength of 450 nm using a microplate reader (BioTek Instruments Inc, Winooski, Vermont, USA) to evaluate cell proliferation.

### 2.3 Transwell

The cells have been digested with trypsin and reintroduced in serum-free media at a density of 5 × 10^5^ cells/mL. Add 200 μL of serum-free DMEM media with 1 × 10^5^ cells (A1896702, Thermo Fisher, Massachusetts, USA) to the Matrigel-coated Transwell chamber, and 500 μL of DMEM medium with 10% FBS (A1896702, Thermo Fisher, Massachusetts, USA) to the bottom chamber. After 48 h of co culturing, the top chamber cells were removed, while the bottom chamber cells were fixed with 4% paraformaldehyde, washed twice with PBS, marked with 0.1% crystal violet solution (198099, Merck, Darmstadt, Germany), and lastly examined and photographed using a Nikon Eclipse E200 microscope (Nikon Corporation, Tokyo, Japan). Quantitative analysis of invasive cells using ImageJ software.

### 2.4 Colony formation experiments

Dilute the cell suspension and inoculate it into a culture dish containing 10 mL of preheated medium at a density of 1000 cells per dish. Gently rotate the dish to ensure even distribution of cells. Subsequently, the culture dish was placed in a cell culture incubator at 37°C, 5% CO_2_, and saturated humidity for 2 weeks. When visible cell clones appear in the culture dish, terminate the culture. Discard the supernatant and add 5 mL of 4% paraformaldehyde (158127, Sigma Aldrich, St. Louis, MO, USA) to fix for 15 min. After fixation, remove the fixative and add 10% diluted Giemsa staining solution (48900, Sigma Aldrich, St. Louis, MO, USA) for 10 min. Subsequently, rinse the dye solution with distilled water and air dry the culture dish. After drying the culture dish, invert it and cover it with a transparent film with a grid. Count the number of colonies containing more than 10 cells to evaluate the cell proliferation ability.

### 2.5 cell scratching assay

Cultivate the cells in a culture dish until they form a dense monolayer of cells. Next, use a sterile pipette tip to scratch a single layer of cells, simulate the wound, and create a “blank” area in the cell layer. Subsequently, the scratched cells were removed, and the media was altered with serum-free DMEM (A1896702, Thermo Fisher, Massachusetts, USA) to ensure that no floating cells reattach to the scratch area. Place the culture dish back into the incubator and observe and photograph the scratched area at 0 h (D0) and 24 h (D1), using a microscope to observe and record cell migration. Measure and compare the width changes of scratch areas at different time points using ImageJ image analysis software (National Institutes of Health, USA) to evaluate the speed of cell migration.

### 2.6 Apoptosis and cell cycle detection

The cell apoptosis experiment was performed using the Annexin V-FITC/PI cell apoptosis detection kit (40302ES60, Yeasen, Shanghai, China) according to the manufacturer’s instructions. Wash the cells twice with PBS after trypsin digestion; Then resuspend the cells in 100 μL of 1 × Binding Buffer, add 5 μL of Annexin V-FITC and 10 μL of PI Staining Solution, and incubate at room temperature in the dark for 15 min; Subsequently, 400 μL of 1 × Binding Buffer was added and detected using CytoFLEX flow cytometry (Beckman Coulter). Use CytExpert software for analysis.

Cell cycle detection was performed using the Cell Cycle and Apoptosis Detection Kit (G1700-50T, Servicebio, Wuhan, China), following the instructions provided. After trypsinizing the cells, the digestion was terminated with serum-containing cell culture medium, followed by a PBS wash. The cells were then fixed in pre-cooled 75% ethanol at 4°C for 2 hours and washed once with PBS. Subsequently, 500 μL of staining solution was added, and the cells were incubated in the dark at 37°C for 30 minutes. Detection was performed using a CytoFLEX flow cytometer, and the data were analyzed using CytExpert software.

### 2.7 Western blot

Total protein in cells was obtained using RIPA lysis buffer (P0013B, Beyotime, Shanghai, China), and protein concentration was measured using BCA protein quantification kit (P0010, Beyotime, Shanghai, China). Protein samples were separated by SDS-PAGE and transferred onto PVDF membranes using a wet transfer method (ab133411, Abcam, Cambridge, UK). The membranes were blocked with 5% non-fat milk (P0216, Beyotime, Shanghai, China). Subsequently, the membranes were incubated overnight at 4°C with primary antibodies specific to the target proteins: OSMR (1:1000, PA5-100298, Thermo Fisher, Massachusetts, USA), JAK (1:500, A7694, Abclonal, Wuhan, China), p-JAK (1:1000, AP0373, Abclonal, Wuhan, China), STAT3 (1:1000, AF1492, Beyotime, Shanghai, China), p-STAT3 (1:1000, GB150001, Servicebio, Wuhan, China), CCL-2 (1:1000, A21991, Abclonal, Wuhan, China), and β-actin (1:1000, ab8227, Abcam, Cambridge, UK). After washing, the membranes were incubated with HRP-conjugated goat anti-rabbit IgG (1:2000, ab6721, Abcam, Cambridge, UK) for 2 h. ECL (A38554, Thermo Fisher, Massachusetts, USA) was used for imaging, and Image J software (V1.8.0.112, NIH, Madison, WI, USA) was used to analyze the bands. β-actin was used as an internal reference to quantitatively analyze the relative expression level of the protein.

### 2.8 Animal modeling and handling

Four week old male BALB/c nude mice were purchased from Fujian Anbuli Biotechnology Co., Ltd. The mice were divided into sh-NC group, sh-OSMR group, sh-OSMR+DMSO group and sh-OSMR+JAKa group, with 6 mice in each group. Adjust the concentration of each group of cells to 5 × 10^7^ cells/mL, and subcutaneously inoculate 0.1 mL on the right abdomen of each mouse. On the 21st day, euthanize the mice, remove the tumor, and measure the volume and weight. Volume calculation formula: V = 1/2 × major axis × minor axis^2^.

### 2.9 RT-qPCR

Extract total RNA from tissues or cells using Trizol (R0016, Beyotime, Shanghai, China). Subsequently, complementary DNA (cDNA) was synthesized using a reverse transcription kit (4368814, Thermo Fisher, Massachusetts, USA). The synthesized cDNA was amplified in a PCR instrument using SYBR Green PCR Master Mix (4309155, Thermo Fisher, Massachusetts, USA) for RT-qPCR. Normalize the relative expression level of the target mRNA to an internal reference (β-actin) using the 2^-ΔΔCt^ formula. The specific primer sequences used in this experiment are shown in **Table 1**.

**Table 1:** RT-qPCR primer sequence.

Name	Species	Primer	Primer sequence
OSMR	Human	F	5’-TCGTGGAGCCCTTCG-3’
		R	5’-GTTCAGCCAAGACTTCACTC-3’
CD206	Human	F	5’-TGCCAGATACAAAAAGGACA-3’
		R	5’-TAACCCACCCATCTTCAGTA-3’
CD163	Human	F	5’-AAAGAAGCAGAGTTTGGTCA-3’
		R	5’-AGGTATCTTAAAGGCTCACTG-3’
IL-10	Human	F	5’-AAAACCAAACCACAAGACAGAC-3’
		R	5’-AGATGCCTTTCTCTTGGAGCTTA-3’
β-actin	Human	F	5’-CACCATTGGCAATGAGCGGTTC-3’
		R	5’-AGGTCTTTGCGGATGTCCACGT-3’
CD206	Mouse	F	5’-TTATGAAAGGCAAGGATGGAT-3’
		R	5’-TCACAACTCAAAACATCCCA-3’
CD163	Mouse	F	5’-GGCTAGACGAAGTCATCTGCAC-3’
		R	5’-CTTCGTTGGTCAGCCTCAGAGA-3’
IL-10	Mouse	F	5’-GCTGGACAACATACTGCTAA-3’
		R	5’-CACCTTGGTCTTGGAGCTTATT-3’
β-actin	Mouse	F	5’-GCCTTCCTTCTTGGGTATGG-3’
		R	5’-GTAAAACGCAGCTCAGTAACA-3’

### 2.10 Flow cytometry analysis of M2 polarization in macrophages

To determine the M2 polarization level of macrophages co cultured with glioblastoma cells and macrophages in mouse tumor tissues, CytoFLEX flow cytometry was used for multi-color flow cytometry analysis.

For tissue processing, tumor tissues were minced into small pieces and digested with DNase (90083, Thermo Fisher, Massachusetts, USA), collagenase II (17101015, Thermo Fisher, Massachusetts, USA), and collagenase IV (17104019, Thermo Fisher, Massachusetts, USA). After passed through a 300-mesh sieve twice, it was treated with red blood cell lysis buffer (C3702, Beyotime, Shanghai, China) and resuspended into a single-cell suspension. Subsequently, F4/80 positive cells were sorted from the single-cell suspension using F4/80 ultra-pure magnetic beads (92-01-0176, Xinxiebio, Suzhou, China). Finally, the sorted cells were stained with CD163 (12-1631-82, Thermo Fisher, Massachusetts, USA) antibody at 4°C and in the dark for 30 min. Wash twice after staining and resuspend in 500 μL PBS. Quantify the percentage of CD163 positive cells using CytoFLEX flow cytometry.

For cells, the co-cultured macrophages were digested with trypsin to prepare a single-cell suspension. Then, the cells were incubated with CD163 (12-1639-42, Thermo Fisher, Massachusetts, USA) antibody at 4°C in the dark for 30 min. After washing with PBS, the percentage of CD163 positive cells was detected using CytoFLEX flow cytometry.

### 2.11 Statistical analysis

Statistical analysis was performed using GraphPad Prism9 (Dotmatics, Boston, MA, USA) software, and the data were expressed as mean ± SD. The detection between two groups was analyzed using t-test, and the comparison between multiple groups was analyzed using one-way ANOVA; *Post hoc* testing was conducted using Tukey’s method, and p < 0.05 was considered statistically significant. Each experiment was repeated at least three times.

## 3 Results

### 3.1 Knocking down OSMR inhibits malignant behavior of tumor cells

**Figure 1:**
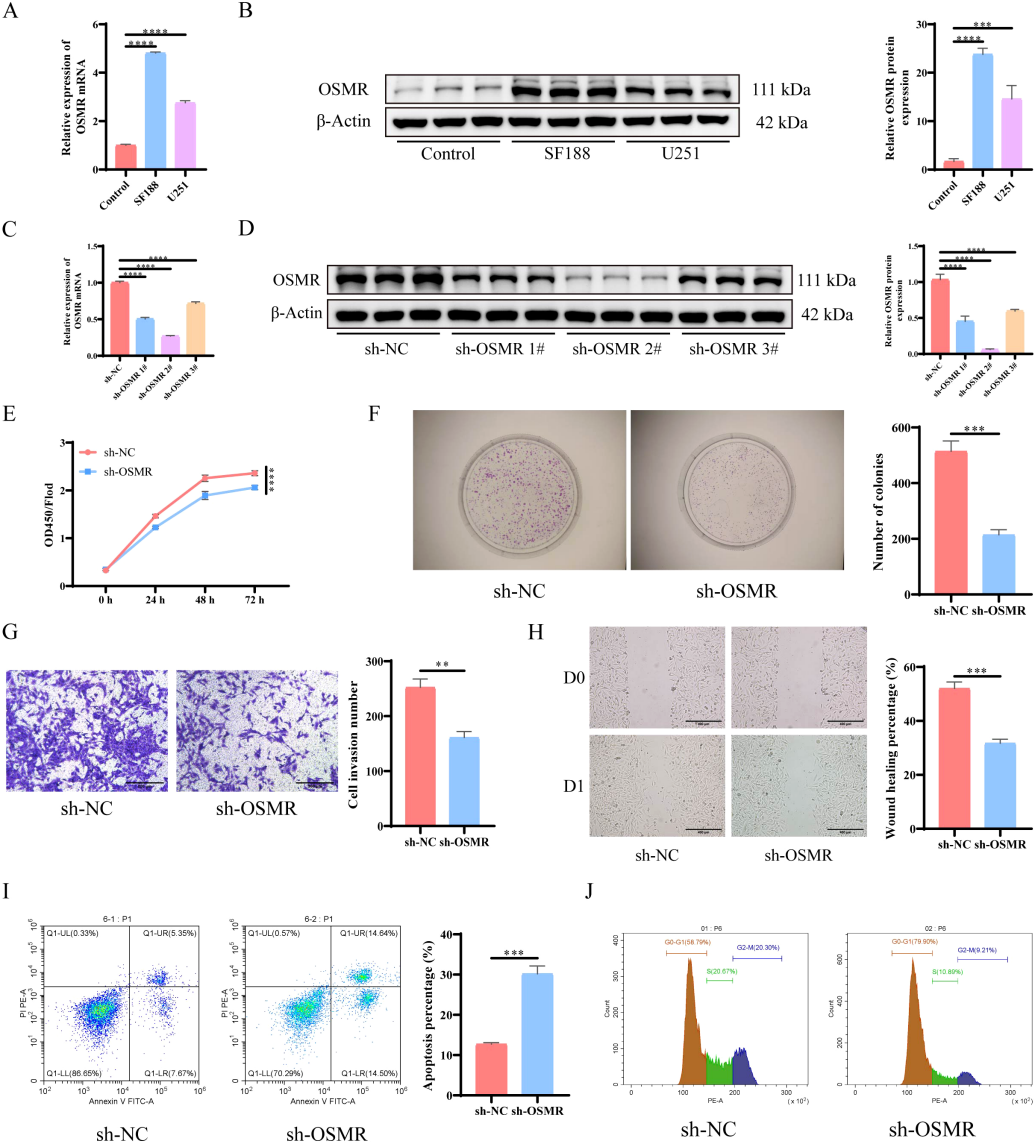
Knocking down OSMR inhibits malignant behavior of tumor cells. **(A)** RT-qPCR was used to detect the expression of OSMR in cells; **(B)** WB detection of OSMR expression in cells; **(C)** RT-qPCR was used to detect the expression of OSMR in cells; **(D)** WB detection of OSMR expression in cells; **(E)** CCK-8 detection of cell proliferation; **(F)** Colony formation experiment to detect cell proliferation; **(G)** Transwell detects cell invasion ability; **(H)** Scratch test to detect cell migration ability; **(I-J)** Flow cytometry is used to detect cell apoptosis rate **(I)** and cell cycle **(J)**. N = 3, **p < 0.01, ***p < 0.001, ****p < 0.0001.

The results of RT-qPCR and WB detection revealed that, when compared to normal astrocytes, OSMR expression levels were significantly increased in both SF188 and U251 cell lines, with SF188 cells exhibiting the greatest expression level (**Figures 1A, B**). Therefore, the SF188 cell line was selected for subsequent experiments. Subsequently, OSMR knockdown plasmids were screened, and the results showed that sh-OSMR 2# had the highest knockdown efficiency on OSMR in the SF188 cell line (**Figures 1C, D**). Subsequent experiments were conducted using sh-OSMR 2#. The experimental results showed that when compared to the sh-NC group, the proliferation ability (**Figures 1E, F**), invasion ability (**Figure 1G**), and migration ability (**Figure 1H**) of tumor cells in the sh-OSMR group were significantly reduced, the apoptosis rate was significantly increased (**Figure 1I**), and the proportion of cell cycle arrest in the G1/G0 phase was significantly increased (**Figure 1J**). These results indicate that knocking down OSMR significantly inhibits the migration, invasion, and proliferation ability of tumor cells, while promoting tumor cell apoptosis and inducing cell cycle arrest.

### 3.2 Knockdown of OSMR inhibits activation of JAK/STAT3/CCL-2 signaling pathway in tumor cells

**Figure 2:**
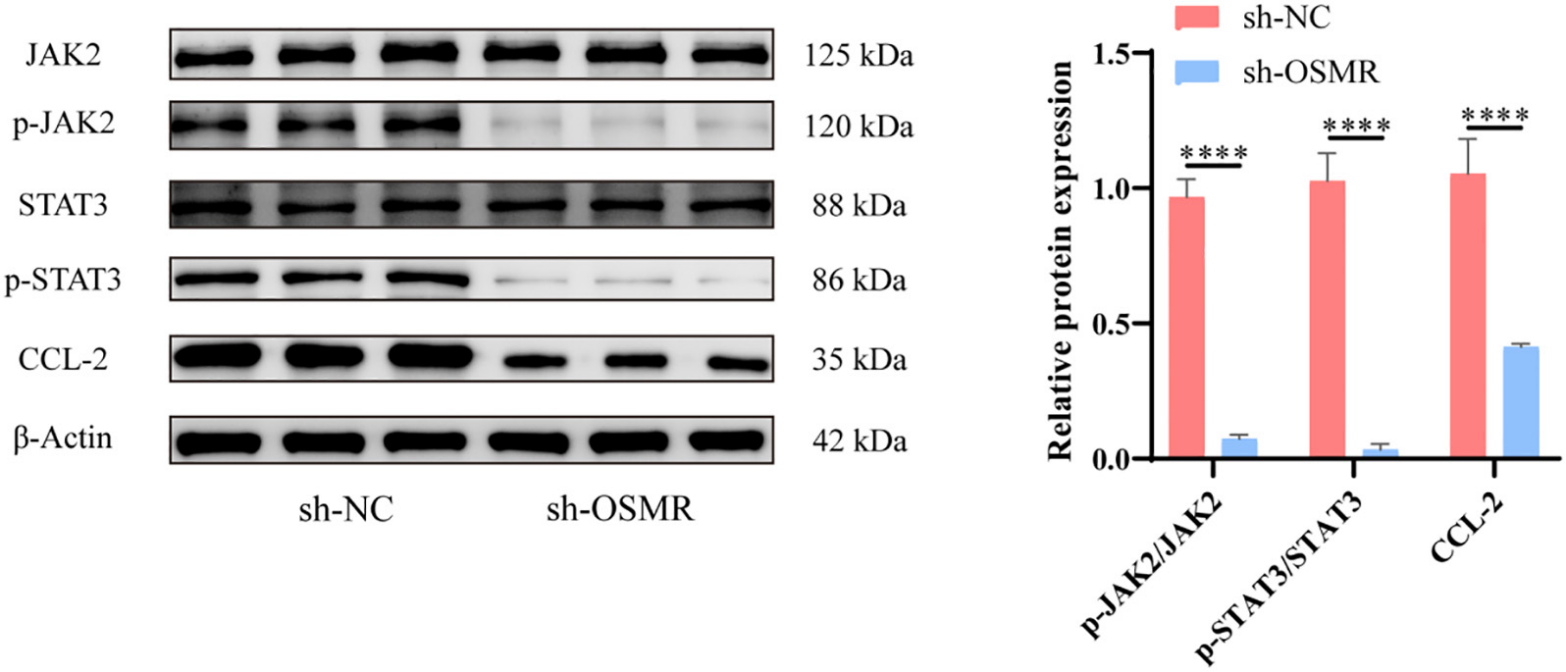
Knockdown of OSMR inhibits activation of JAK/STAT3/CCL-2 signaling pathway in tumor cells. WB detection of JAK, p-JAK, STAT3, p-STAT3 and CCL-2 expression in cells and quantitative analysis. N = 3, ****p < 0.0001.

The WB test results revealed that the expression levels of JAK/STAT3/CCL-2 pathway-related proteins p-JAK/JAK, p-STAT3/STAT3 and CCL-2 were significantly cut down in the tumor cells of the sh-OSMR group relative to the sh-NC group (**Figure 2**). It was discovered that OSMR knockdown suppressed the activation of JAK and STAT3 as well as the expression of CCL-2, implying that OSMR silencing could inhibit the stimulation of the JAK/STAT3/CCL-2 signaling pathway in tumor cells, potentially inhibiting the malignant behaviors of glioblastoma cells by regulating this pathway.

### 3.3 OSMR activates JAK/STAT3/CCL-2 pathway to promote malignant behavior of tumor cells

**Figure 3:**
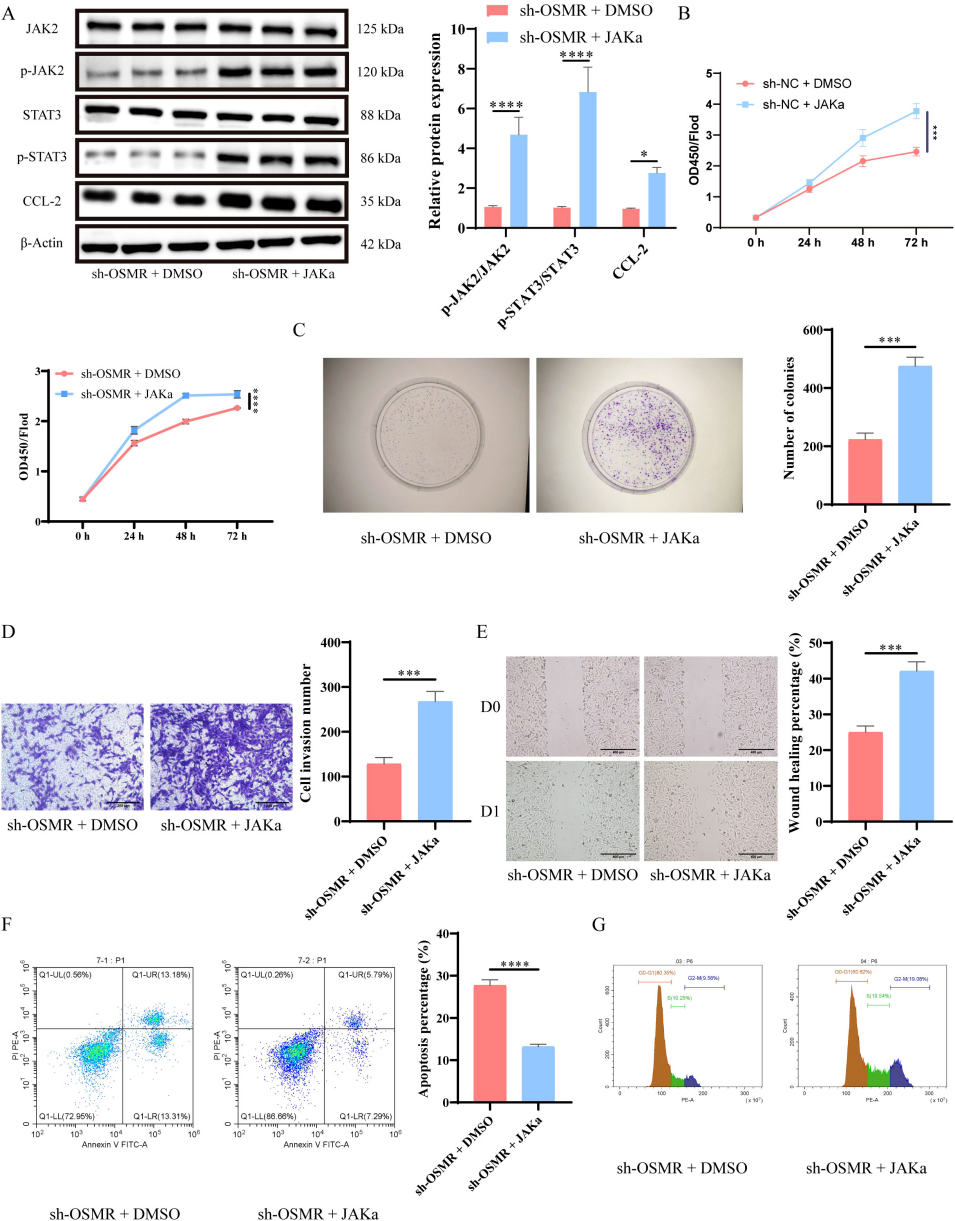
OSMR activates JAK/STAT3/CCL-2 pathway to promote malignant behavior of tumor cells. **(A)** WB detection of JAK, p-JAK, STAT3, p-STAT3 and CCL-2 expression in cells and quantitative analysis; **(B)** CCK-8 detection of cell proliferation; **(C)** Colony formation experiment to detect cell proliferation; **(D)** Transwell detects cell invasion ability; **(E)** Scratch test to detect cell migration ability; **(F-G)** Flow cytometry is used to detect cell apoptosis rate **(F)** and cell cycle **(G)**. N = 3, ***p < 0.001, ****p < 0.0001.

WB analysis showed that after JAKα intervention, the phosphorylation of JAK and STAT3, as well as the expression of CCL-2, were significantly increased (**Figure 3A**). CCK-8 detection and establishment of colonies studies revealed that tumor cells in the sh-OSMR + JAKa group proliferated substantially more than those in the sh-OSMR + DMSO group (**Figures 3B, C**). The Transwell experiment results demonstrated that contrasted with the sh-OSMR+DMSO group, the invasion ability of tumor cells in the sh-OSMR+JAKa group was much better (**Figure 3D**). The scratch test results revealed that compared to the sh-OSMR+DMSO group, the migration ability of tumor cells in the sh-OSMR+JAKa group was significantly enhanced (**Figure 3E**). Theta flow cytometry results also demonstrated that the mortality rate of tumor cells in the sh-OSMR+JAKa group was dramatically reduced (**Figure 3F**), as well as the fraction of G1/G0 phase cells (**Figure 3G**). These results indicate that JAK agonists can reverse the inhibitory effect of OSMR silencing on malignant tumor cell behavior, indicating that OSMR enhances aggressive behavior by activating the JAK/STAT3/CCL-2 pathway.

### 3.4 OSMR/JAK/STAT3/CCL-2 pathway regulates malignant behavior of tumor cells and induces M2 polarization of macrophages

**Figure 4:**
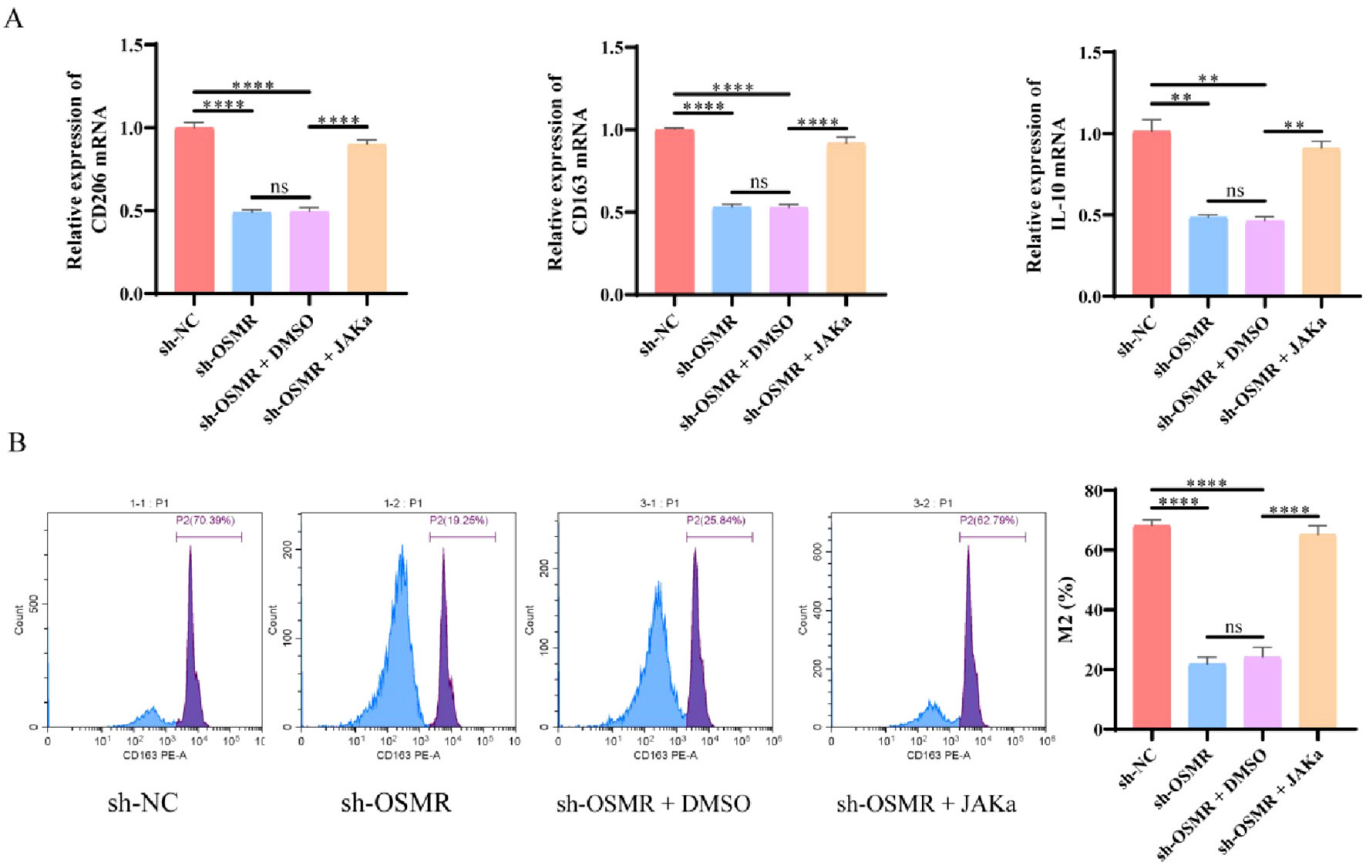
OSMR/JAK/STAT3/CCL-2 pathway regulates malignant behavior of tumor cells and induces M2 polarization of macrophages. **(A)** RT-qPCR was used to detect the expression of M2 phenotype marker genes (CD206, CD163, IL-10) in co cultured macrophages; **(B)** Flow cytometry was used to detect the percentage of M2 macrophages in co cultured macrophages. N = 3, ns p > 0.05, **p < 0.01, ****p < 0.0001.

To further investigate the effect of OSMR mediated JAK/STAT3/CCL-2 pathway regulation on malignant behavior of GBM cells and macrophage polarization, we co cultured SF188 cells with THP-1 generated microglia to simulate the tumor microenvironment. RT-qPCR data revealed that, when compared to the sh-NC group, the transcript levels of CD206, CD163, and IL-10 in macrophages of the sh-OSMR group and sh-OSMR+DMSO group were significantly lower, and the addition of JAK agonists was able to reverse these changes (**Figure 4A**). The flow cytometry facts revealed that, when compared to the sh-NC group, the percentage of M2 macrophages dropped in the sh-OSMR group and the sh-OSMR+DMSO group, whereas the proportion of M2 monocytes increased substantially with the addition of JAK agonists (**Figure 4B**). These results indicate that knocking down OSMR decreases M2 polarization in co cultured macrophages, while the use of JAK agonists can reverse this effect. In summary, OSMR activation of the JAK/STAT3/CCL-2 pathway promotes malignant behavior of GBM cells and induces M2 polarization in macrophages.

### 3.5 OSMR activates JAK/STAT3/CCL-2 pathway to promote tumor growth and M2 polarization of macrophages

**Figure 5:**
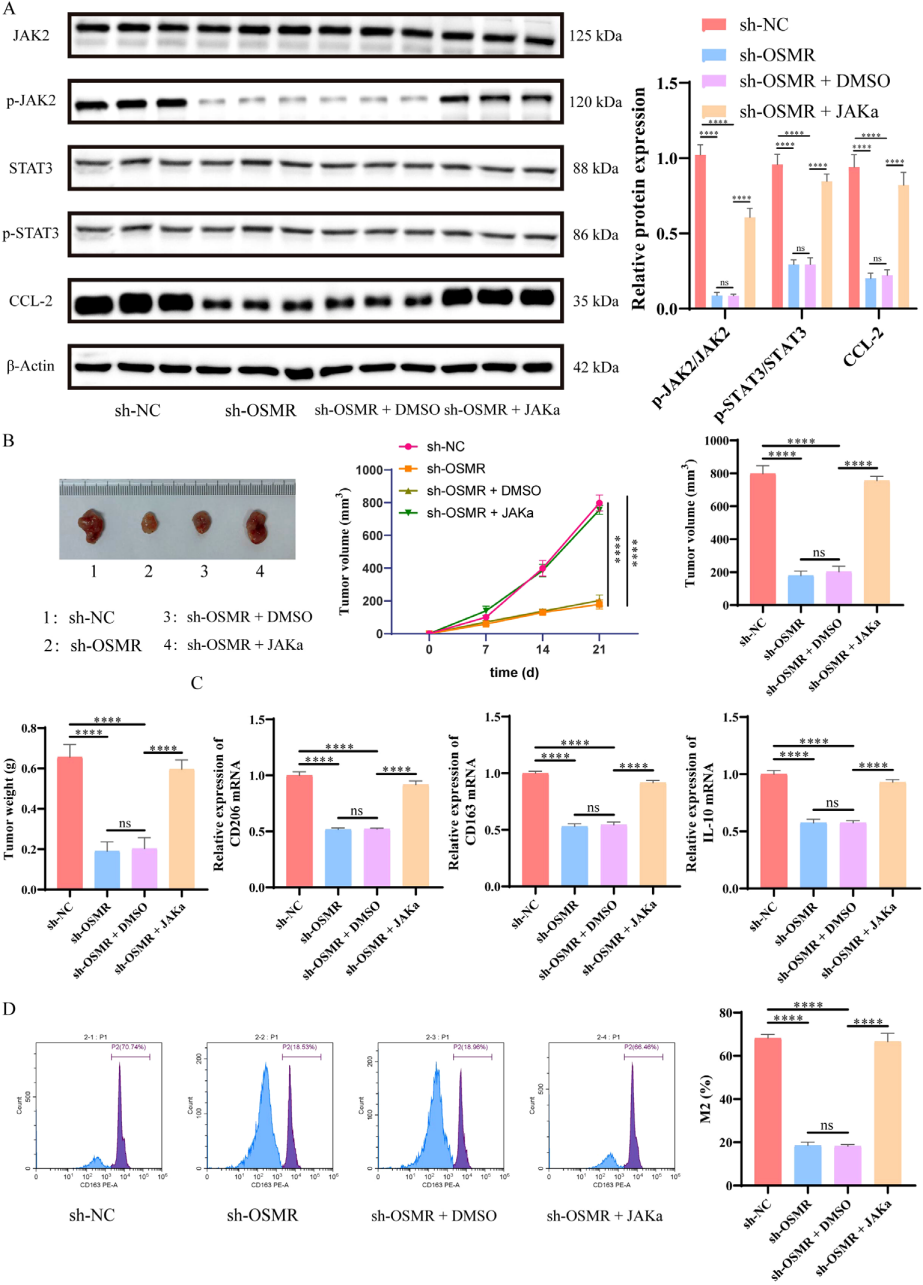
OSMR activates JAK/STAT3/CCL-2 pathway to promote tumor growth and M2 polarization of macrophages. **(A)** WB detection of JAK, p-JAK, STAT3, p-STAT3 and CCL-2 expression in cells and quantitative analysis. **(B)** Images, tumor volume, and weight of subcutaneous tumors in each group of mice; **(C)** RT-qPCR was used to detect the expression of M2 phenotype marker genes (CD206, CD163, IL-10) in macrophages in tumor tissues; **(D)** Flow cytometry is used to detect the percentage of M2 macrophages in tumor tissue macrophages. N = 6, ns p > 0.05, ****p < 0.0001.

The WB analysis showed that in *in vivo* experiments, JAKα intervention could reverse the inhibition of JAK and STAT3 phosphorylation, as well as the suppression of CCL-2 caused by OSMR knockdown (**Figure 5A**). Furthermore, as compared to the sh-NC group, the tumor volume and weight of the sh-OSMR group and the sh-OSMR+DMSO group mice were significantly reduced, although JAK agonists were able to counteract the inhibitory effect of sh-OSMR (**Figure 5B**); The results of RT-qPCR indicated that the transcript levels of CD206, CD163, and IL-10 in the tumor tissues of mice in the sh-OSMR group and sh-OSMR+DMSO group were significantly reduced compared with the sh-NC group, while JAK agonist treatment could restore the expression of these mRNAs (**Figure 5C**); Flow cytometry analysis revealed that the percentage of M2 macrophages in tumor tissues of mice in the sh-OSMR group and sh-OSMR+DMSO group decreased, while treatment with JAK agonists offset this change (**Figure 5D**). These findings suggest that OSMR enhances tumor growth and macrophage M2 polarization via activating the JAK/STAT3/CCL-2 pathway.

## 4 Discussion

Gliomas are the most prevalent primary cerebral malignant tumors in adults. Due to their strong proliferative capacity and invasiveness, surgical resection alone is difficult to cure. Even with combination chemotherapy, there are still many poor prognoses and extremely high recurrence rates (30). Therefore, finding new treatment options is urgent and important.

OSMR is a cell surface protein that plays a crucial role in several physiological processes, is highly expressed in various malignancies, and regulates the proliferation and invasion of cancer cells (16, 18). Studies have indicated that OSMR expression increases dramatically in GBM and has a role in regulating GBM invasion (31). Therefore, we performed OSMR knockdown treatment on GBM and detected the functional changes of GBM after OSMR knockdown. The detection results showed that after knocking down OSMR, GBM migration, invasion, and proliferation abilities of were dramatically reduced, and cellular death rate was significantly increased. The volume and weight of sh-OSMR tumor tissue *in vivo* were significantly reduced. This is similar to how OSMR acts on cancer cells in cervical cancer (32) and ovarian cancer (33), showing that OSMR can increase the growth and development of GBM.

Previous research has revealed that OSMR’s participation in cancer is strongly tied to the JAK/STAT3 signaling pathway (24), and that inhibiting the STAT3/CCL2 signaling pathway may inhibit tumor malignancy (34). In this investigation, we also discovered that suppressing OSMR expression significantly reduced JAK and STAT3 phosphorylation levels of difficulty, as well as the presentation of their downstream component CCL-2 in GBM. However, the addition of a JAK agonist reversed these changes and counteracted the inhibitory effect of OSMR knockdown on GBM malignant behaviors. It is worth noting that JAK agonists, while activating the JAK-STAT pathway, may also exert their effects by promoting the activation of the MAPK and PI3K pathways (29). Therefore, future studies should further validate the targeting specificity of Butyzamide and explore its specific role in the interaction of multiple signaling pathways.

TAM is a key component of the tumor microenvironment and plays a significant role in cancer progression (9). The M2 polarization of macrophages has a positive significance for the development of cancer (13). M2-like polarized macrophages play an important role in promoting the progression of GBM (35), and the polarization of M2 macrophages has been shown to support glioma growth (36). While IL-10, CD206, CCL17, CCL18, PPARγ, CCL22, TGF-β, and MMP9 are all markers of M2 macrophage polarization, CD206 and IL-10 are commonly used as the most reliable indicators for detecting M2 macrophage polarization (37). Moreover, CD163 is considered one of the most significant markers for the activation of M2 macrophages (38). The detection of these markers can effectively reflect the activation status of M2 macrophages. Our cell experiment results showed that knocking down OSMR significantly reduced the transcript levels of M2 polarization-related factors CD206, CD163, and IL-10 in monocytes co cultured with GBM cells, while also decreasing the proportion of M2 macrophages. This indicates that knocking down OSMR regulates the malignant behavior of GBM cells and inhibits M2 polarization of macrophages, while the use of JAK agonists reverses this effect. In *in vivo* experiments, we further validated this discovery.

Although we have confirmed in cell and animal experiments that OSMR promotes TAM M2 polarization through the JAK/STAT3/CCL-2 signaling pathway. However, there are still some shortcomings in this study, which failed to verify whether overexpression of OSMR would lead to further development of GBM. In addition, although we strictly controlled the experimental conditions, the results of the CCK-8 assay were relatively mild, which may be due to subtle variations in the culture conditions. While the current results are sufficient to support our conclusions, this also indicates that there is still room for further optimization of our experiments to improve the significance and reliability of the results. In summary, we have demonstrated that OSMR induces M2 polarization of TAMs *in vitro* and *in vivo* via the JAK/STAT3/CCL-2 signaling pathway.

## 5 Conclusion

OSMR regulates the development of GBM and M2 polarization of TAM through the JAK/STAT3 signaling pathway.

## Data Availability

The original contributions presented in the study are included in the article/**Supplementary Material**. Further inquiries can be directed to the corresponding author/s.
