# Supplementary material for: OSMR induces M2 polarization of glioblastoma associated macrophages through JAK/STAT3 signaling pathway
Source: Front Oncol. 2025 Mar 14;15:1538649. doi: 10.3389/fonc.2025.1538649 (PMC11949811; doi:10.3389/fonc.2025.1538649)
Supplement: Supplementary file 1 [file DataSheet1.pdf]

Figure 1B. Original Western Blot.

OSMR 111 kDa

β-Actin 42 kDa

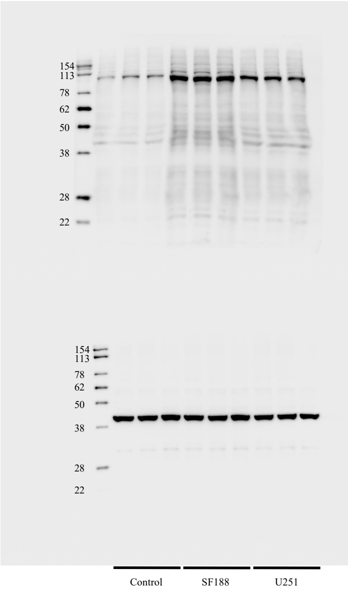

Figure 1B shows the whole blot after cutting membrane at molecular weight 111 kDa and 42 kDa for OSMR (111 kDa) and β-Actin (42 kDa).

Figure 1D. Original Western Blot.

OSMR 111 kDa

β-Actin 42 kDa

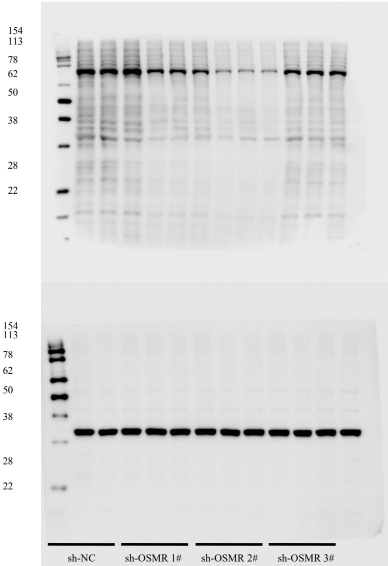

Figure 1D shows the whole blot after cutting membrane at molecular weight 111 kDa and 42 kDa for OSMR (111 kDa) and β-Actin (42 kDa).

Marker: G2086 Servicebio

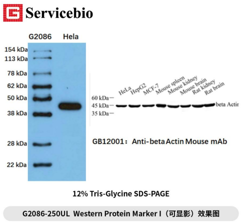

Figure 2A. Original Western Blot.

JAK2 125 kDa

p-JAK2 120 kDa

STAT3 88 kDa

p-STAT3 86 kDa

CCL-2 35 kDa

β-Actin 42 kDa

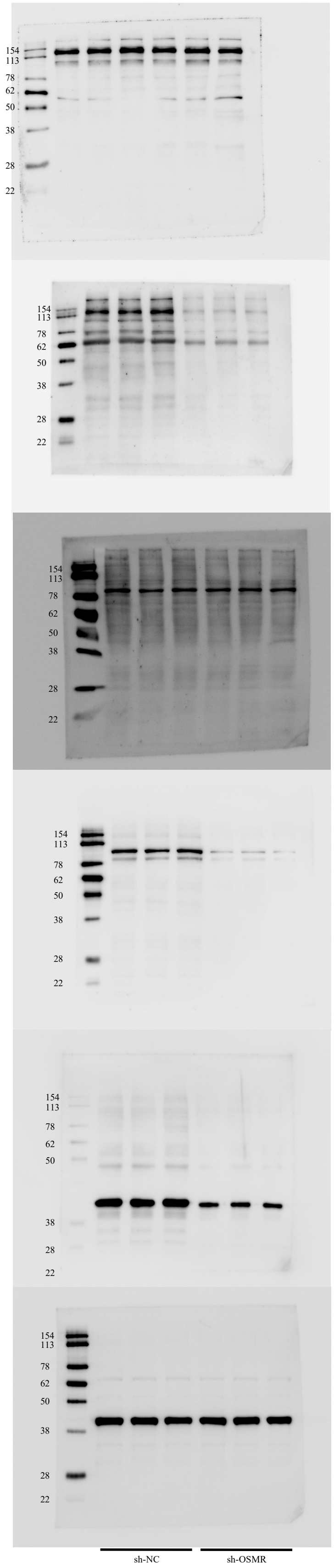

Figure 2A shows the whole blot after cutting membrane at molecular weight 125 kDa, 120 kDa, 88 kDa, 86 kDa, 35 kDa and 42 kDa for JAK2 (125 kDa), p-JAK2 (120 kDa), STAT3 (88 kDa), p-STAT3 (86 kDa), CCL-2 (35 kDa) and β-Actin (42 kDa).

Marker: G2086 Servicebio

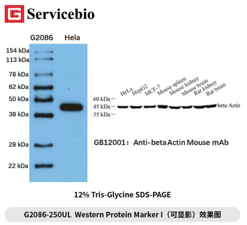

Figure 3A. Original Western Blot.

JAK2 125kDa

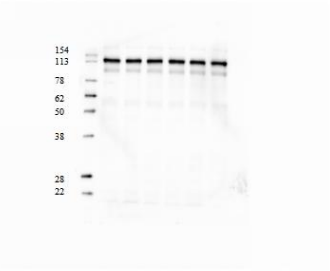

p-JAK2 120kDa

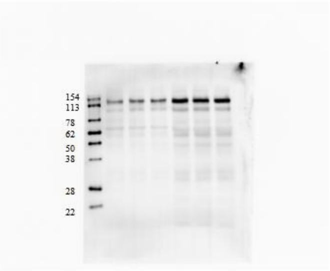

Figure 3A shows the whole blot after cutting membrane at molecular weight 125 kDa, 120 kDa, 88 kDa, 86 kDa, 35 kDa and 42 kDa for JAK2 (125 kDa), p-JAK2 (120 kDa), STAT3 (88 kDa), p-STAT3 (86 kDa), CCL-2 (35 kDa) and β-Actin (42 kDa).

STAT3 88kDa

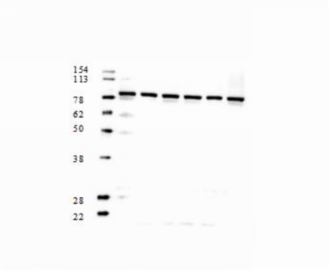

p-STAT3 86kDa

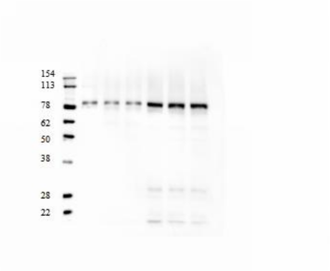

Marker: G2086 Servicebio

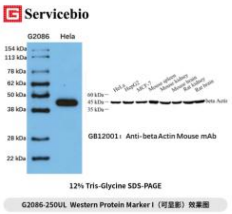

CCL-2 35kDa

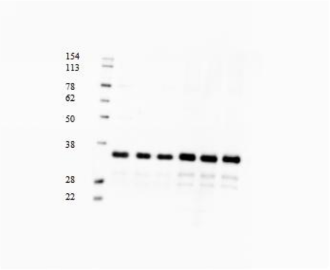

β-Actin 42kDa

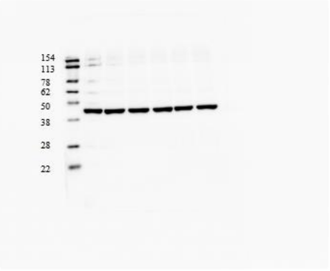

Figure 5A. Original Western Blot.

JAK2 125kDa

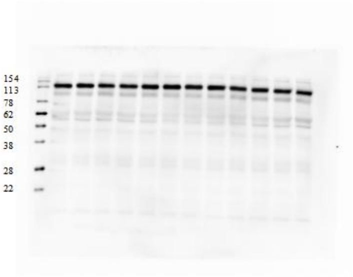

p-JAK2 120kDa

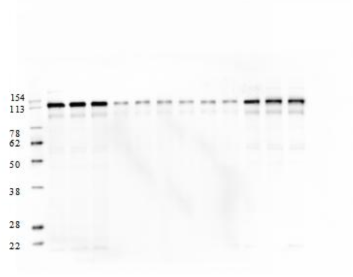

Figure 5A shows the whole blot after cutting membrane at molecular weight 125 kDa, 120 kDa, 88 kDa, 86 kDa, 35 kDa and 42 kDa for JAK2 (125 kDa), p-JAK2 (120 kDa), STAT3 (88 kDa), p-STAT3 (86 kDa), CCL-2 (35 kDa) and β-Actin (42 kDa).

STAT3 88kDa

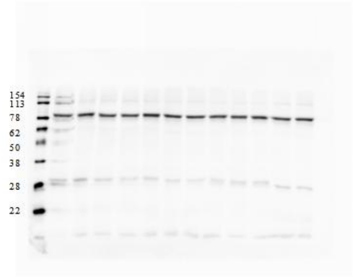

p-STAT3 86kDa

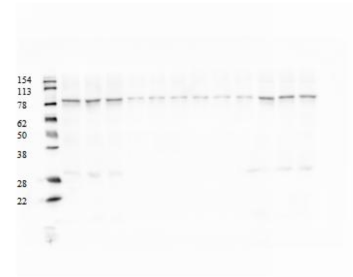

CCL-2 35kDa

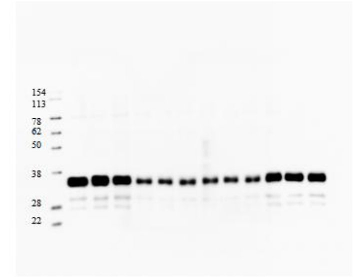

Marker: G2086 Servicebio

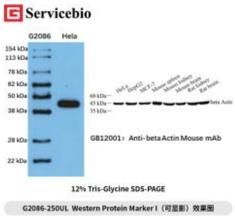

β-Actin 42kDa

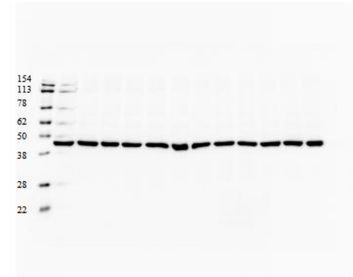

Figure 1F. Colony formation experiment.

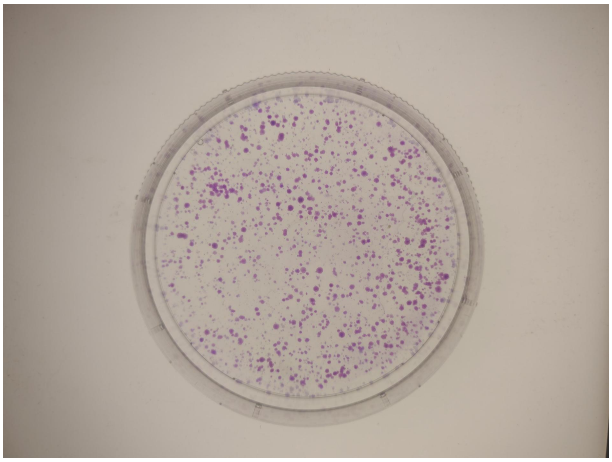

sh-NC

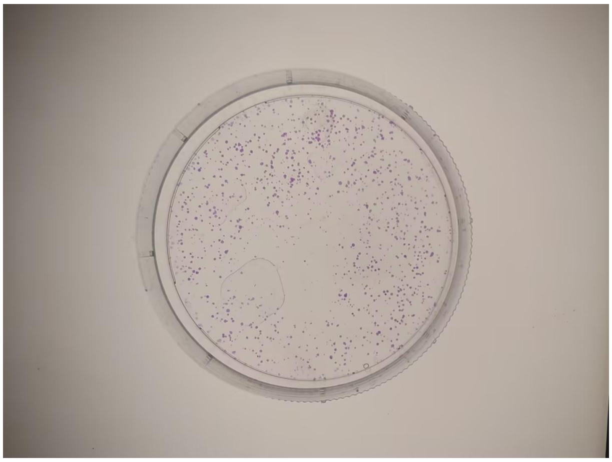

sh-OSMR

Figure 3B. Colony formation experiment.

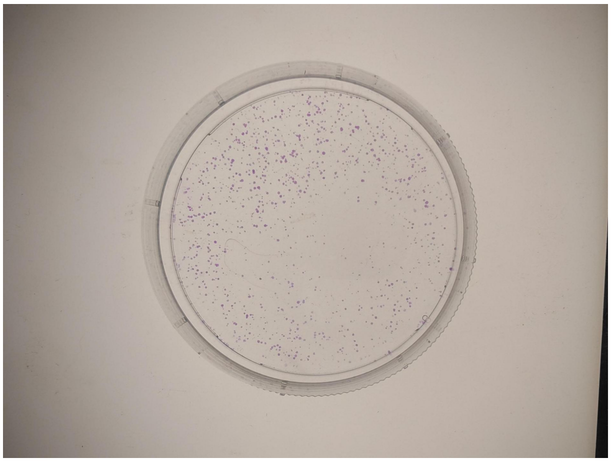

sh-OSMR + DMSO

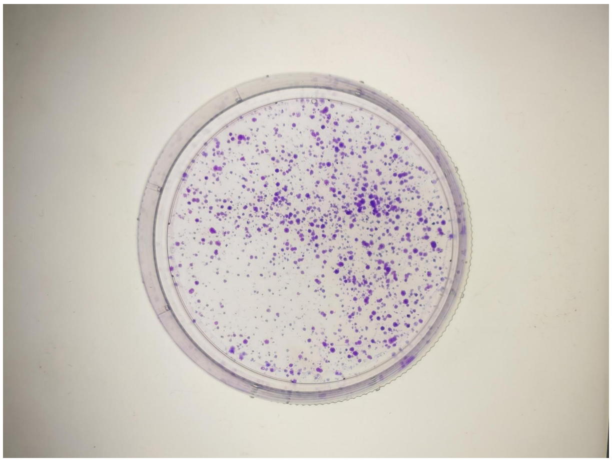

sh-OSMR + JAKa

Figure 1G. Transwell.

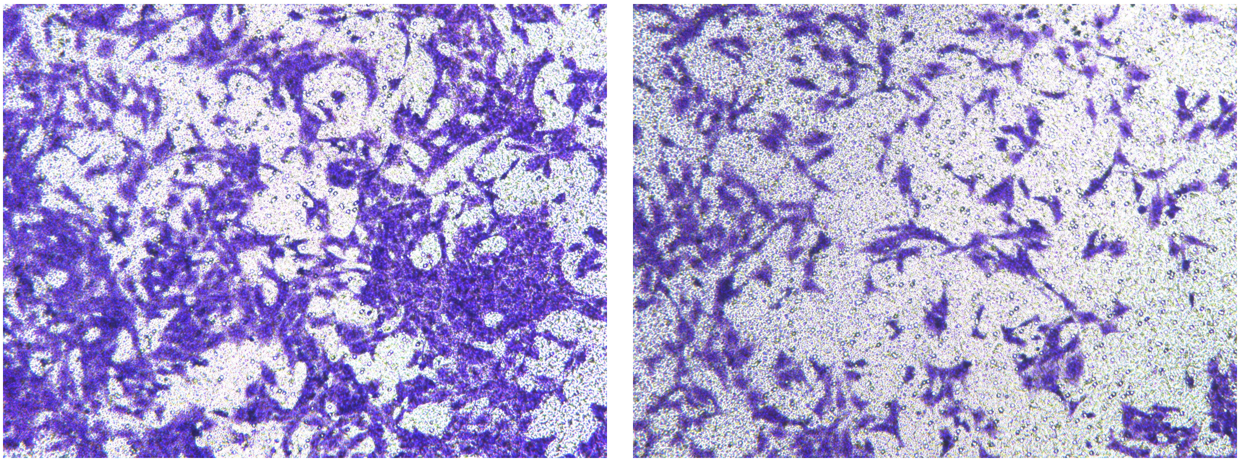

sh-NC   sh-OSMR

Figure 3C. Transwell.

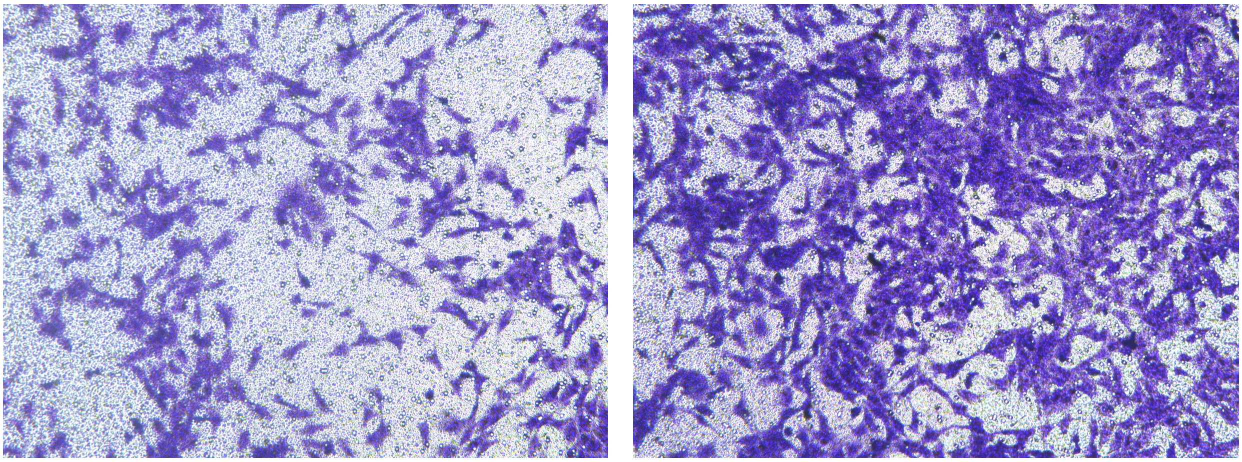

sh-OSMR + DMSO   sh-OSMR + JAKa

Figure 1H. Scratch test.

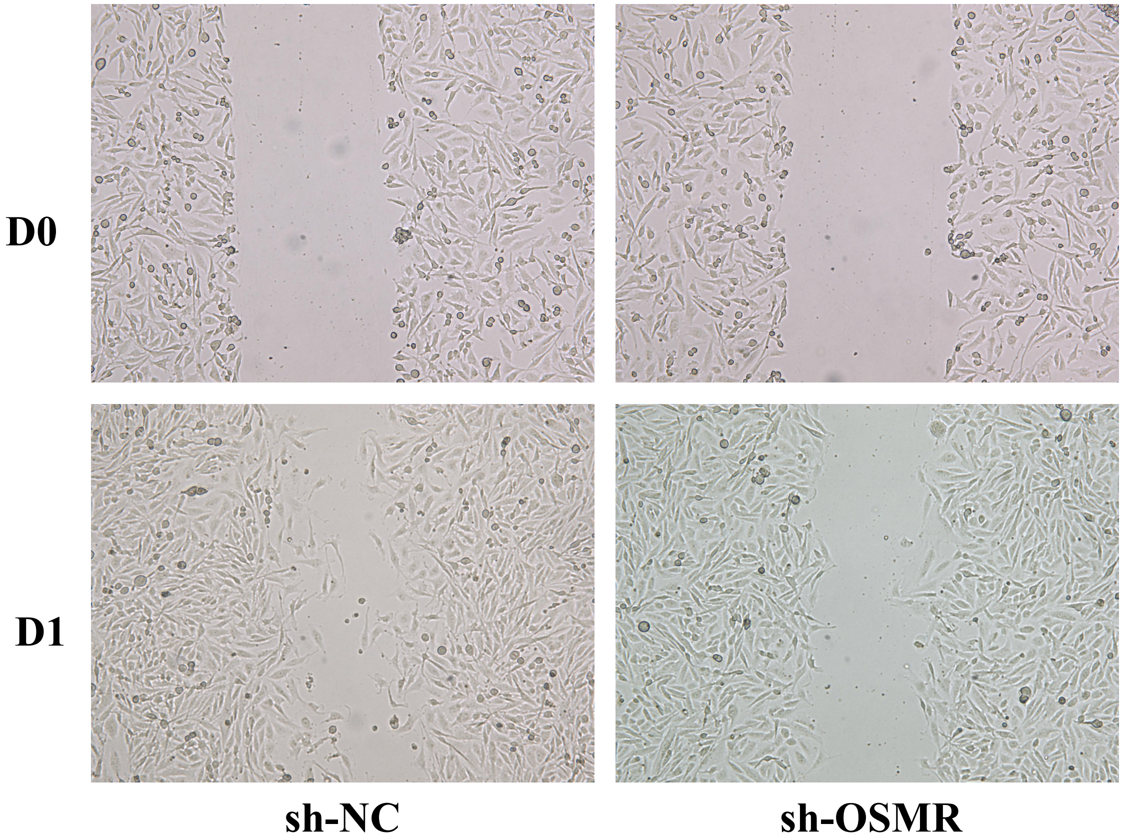

Figure 3D. Scratch test.

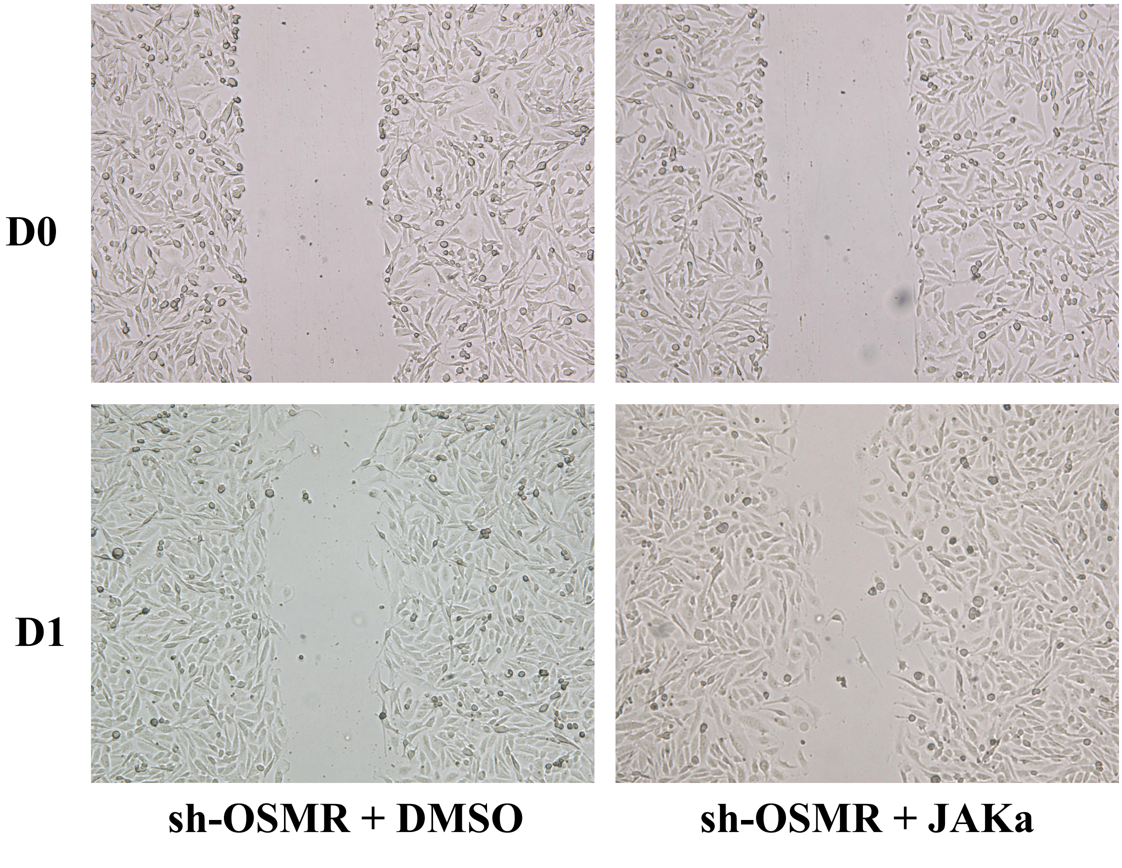

Flow cytometry

Apoptosis

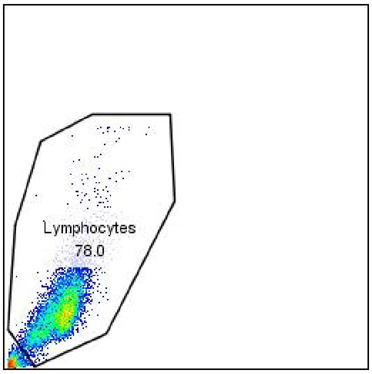

6-1.fcs
Ungated
Pseudocolor of FSC-H() vs. SSC-H()
100
10000
6-1.fcs

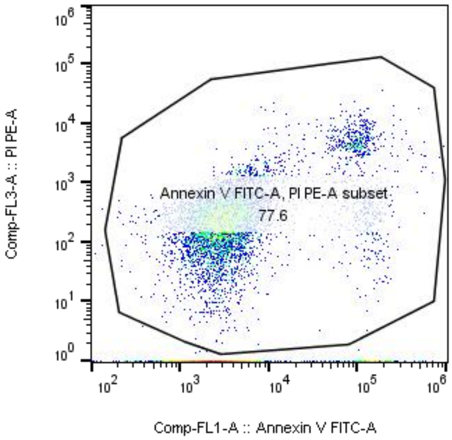

6-1.fcs
Lymphocytes
Pseudocolor of Comp-FL1-A() vs. Comp-FL3-A()
78.0
7795
6-1.fcs/Lymphocytes

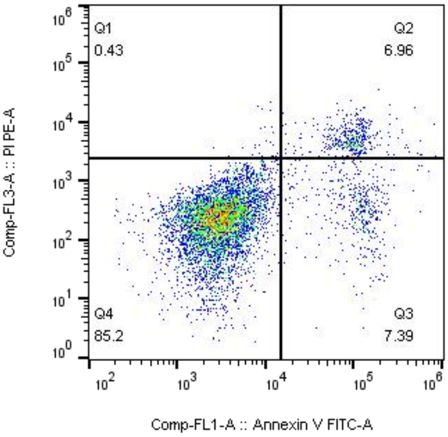

6-1.fcs
Annexin V FITC-A, PI PE-A subset
Pseudocolor of Comp-FL1-A() vs. Comp-FL3-A()
60.5
6048
6-1.fcs/Lymphocytes/Annexin V FITC-A, PI PE-A subset

M2 polarization level of macrophages

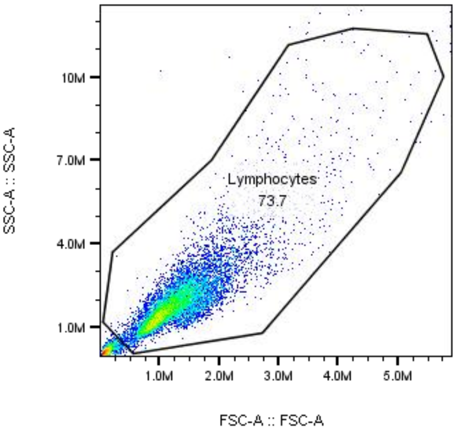

1-1.fcs
Ungated
Pseudocolor of FSC-A() vs. SSC-A()
100
14396
1-1.fcs

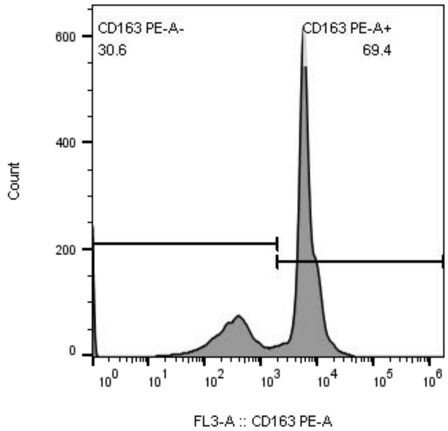

1-1.fcs
Lymphocytes
Histogram* of FL3-A()
73.7
10607
1-1.fcs/Lymphocytes
