# Supplementary figures and images for: OSMR induces M2 polarization of glioblastoma associated macrophages through JAK/STAT3 signaling pathway
Source: Front Oncol. 2025 Mar 14;15:1538649. doi: 10.3389/fonc.2025.1538649 (PMC11949811; doi:10.3389/fonc.2025.1538649)

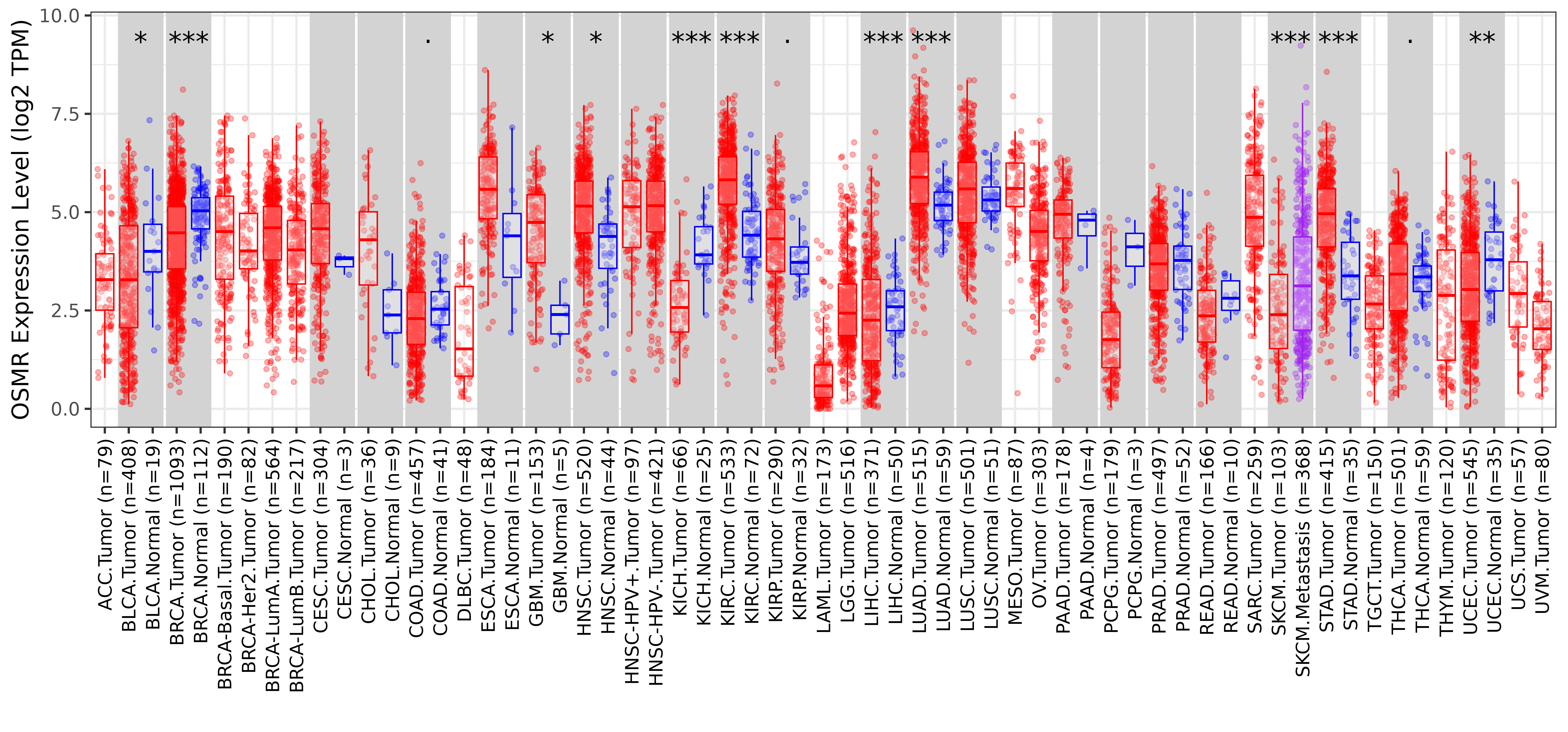

Supplement: Supplementary file 2 [file Image1.jpeg]
